# Trastuzumab effects depend on HER2 phosphorylation in HER2-negative breast cancer cell lines

**DOI:** 10.1371/journal.pone.0234991

**Published:** 2020-06-25

**Authors:** Anna Burguin, Daniela Furrer, Geneviève Ouellette, Simon Jacob, Caroline Diorio, Francine Durocher

**Affiliations:** 1 Centre de recherche sur le cancer, Centre de recherche du CHU de Québec-Université Laval, Québec, Canada; 2 Département de médecine moléculaire, Faculté de médecine, Université Laval, Québec, Canada; 3 Département de médecine sociale et préventive, Faculté de médecine, Université Laval, Québec, Canada; 4 Laboratoire de pathologie, Hôpital du Saint-Sacrement, CHU de Québec-Université Laval, Québec, Canada; 5 Centre des Maladies du Sein, Hôpital du Saint-Sacrement, Québec, Canada; IMDEA Food Institute (CEI-UAM+CSIC), SPAIN

## Abstract

The breast cancer (BC) biomarker HER2 (Human Epidermal Receptor 2) is overexpressed in 25% of BC. Only patients with HER2-positive tumors receive HER2-targeting therapies, like trastuzumab (Herceptin). However, some women with a HER2-negative BC could benefit from trastuzumab. This could be explained by the activation/phosphorylation of HER2 that can be recognized by trastuzumab. The aim of this study is to examine trastuzumab effects on HER2 phosphorylation at tyrosine Y877 (pHER2^Y877^). HER2 and pHER2^Y877^ status were evaluated in a cohort of BC patients representative of molecular subtypes distribution (n = 497) and in a series of BC cell lines (n = 7). Immunohistochemistry against pHER2^Y877^ was performed on tissue micro arrays. Cellular proliferation assays were performed on BC cell lines presenting different combinations of HER2 and pHER2^Y877^ status and treated with increasing doses of trastuzumab (0–150 μg/ml). The prevalence of pHER2^Y877^ in this cohort was 6%. Nearly 5% of patients with HER2-negative tumors (n = 406, 82%) overexpressed pHER2^Y877^. Among triple negative BC patients (n = 39, 8%), 7.7% expressed pHER2^Y877^. Trastuzumab treatment decreased cell proliferation in HER2−/pHER2^Y877^+ BC cell lines, to an extent comparable to what occurs in HER2+ cell lines, but did not affect HER2−/pHER2^Y877^− cell lines. Trastuzumab sensitivity in HER2−/pHER2^Y877^+ cell line is specific to HER2 tyrosine 877 phosphorylation. Hence, with further confirmation in a bigger cohort, trastuzumab treatment could be envisaged as a treatment option to women presenting with HER2−/pHER2+ tumors, representing more than 1000 BC women in Canada in 2019.

## Introduction

According to the Public Health Agency of Canada (PHAC), breast cancer (BC) represents 25% of new cases of cancers and 13% of all cancer deaths in women in 2019. Although statistics are provided for one disease, BC can be classified into four molecular subtypes depending on the overexpression of three molecular markers: estrogen receptor (ER), progesterone receptor (PR), and Human Epidermal Receptor 2 (HER2) [1]. These four molecular subtypes are described as follows: Luminal A (ER and/or PR-positive and HER2-negative), Luminal B (ER and/or PR-positive and HER2-positive), HER2 (ER and PR-negative and HER2-positive), and triple negative, also known as TNBC (ER, PR and HER2-negative) [2].

HER2, also known as ErbB2, is part of the HER (EGFR, Epidermal Growth Factor Receptor) family of receptor tyrosine kinases (RTK), along with HER1 (ErbB1), HER3 (ErbB3), and HER4 (ErbB4) [3–6]. They play an important role in cellular functions as they are involved in multiple signaling pathways which mediate cellular proliferation, migration or adhesion [7,8]. HER family members are composed of an extracellular domain involved in ligand binding and dimerization with another HER family member, a unique transmembrane domain, and a catalytic intracellular domain. HER activation occurs by trans-autophosphorylation after dimerization with another HER family member [9,10]. That is, a phosphorylated tyrosine localized in the tyrosine kinase domain of one of the partner will mediate the tyrosine phosphorylation of the other partner and vice versa [11]. HER2 is particular because it can not bind ligands, but it is the receptor preferably chosen for dimerization by the other HER family members.

HER2 is overexpressed in 20 to 25% of BC [12]. It is also known to be a therapeutic target for trastuzumab (Herceptin, Genentech, San Francisco, CA), a monoclonal antibody that binds HER2 extracellular subdomain IV. Trastuzumab was approved by the US Food and Drug Administration in 1998 [13]. So far, its mechanism of action is still not fully understood, but it has been shown to increase progression-free and overall survival (OS) when coupled with chemotherapy or as adjuvant treatment [14,15].

Currently, anti-HER2 agents are only administered to patients with a HER2-positive (HER2+) tumor (protein overexpression (3+) at the immunohistochemistry (IHC) or gene amplified by *in situ* hybridization (ISH)) [16,17]. However, some studies have shown that anti-HER2 therapy actually improved Disease-Free Survival (DFS) and OS in a subgroup of patients who are considered HER2-negative (HER2−) by IHC and fluorescence ISH (FISH) [14,18]. Paik *et al*. reported that in the National Surgical Adjuvant Breast and Bowel Project (NSABP) trial B-31, all patients having tumors identified as “central HER2-negative” and patients that have a non-amplification of *HER2* gene copy number benefited from trastuzumab [14]. An explanation for this result could be that tumors that have an over-phosphorylation of HER2 may also be sensitive to trastuzumab. As mentioned, HER2 activation is driven by its phosphorylation. Hence receptor phosphorylation in C-terminal tyrosines associated with HER2 activation is a critical step in stimulating recruitment of cytoplasmic signal transducers involved in different signaling pathways, such as PI3K/Akt or Raf/MAPK [19]. Those signaling pathways mediate cell adhesion, proliferation, differentiation and migration, and, if deregulated, can lead to cancer [10,20].

Despite more than 30 HER2 phosphorylation (pHER2) sites reported in PhosphoSitePlus®10 and the availability of antibodies directed against at least 11 of these sites, the abundance of pHER2 at only three phosphorylated sites has been analyzed by IHC in BC patients to date, and this by a few studies only. HER2 phosphorylation at Y1221/1222 and Y1248 was evaluated in different BC patients cohorts [21–27]. PHER2+ identified a subgroup of patients with worse prognosis and less survival among untreated patients and a subgroup of patients with a better response among those treated with the anti-HER2 therapies [21,23,25,28]. Studies have reported that Y877 phosphorylation is a marker of HER2 activation. It is also the principal tyrosine phosphorylation site during HER2 activation [29–32]. In addition, Telesco *et al*. have shown that Y877 is sufficient to induce a complete activation of HER2 and that it can maintain its activation conformation [31]. There is no study focusing on Y877. However, previous results confirm that Y877 has a major role in HER2 activation. Thus, is critical to study Y877 in order to improve BC diagnosis and treatment.

Hence, we hypothesize that the over-activation of HER2, reflected by the over-phosphorylation at position Y877 of the receptor, could be recognized by trastuzumab, and that HER2 phosphorylation could be biologically more relevant than simple protein overexpression in BC diagnosis.

In this study, we established for the first time the prevalence of phosphorylated HER2 at position Y877 in a patient cohort representative of BC molecular subtypes, and we analyzed trastuzumab effects depending on HER2 phosphorylation in a series of BC cell lines.

## Materials and methods

### Cohort recruitment and tissue micro array construction

Patients were recruited consecutively between 2011 and 2012 at the Centre des maladies du sein (CMS) de Québec to obtain a representative cohort of the CMS attendees [33]. For this cohort, 558 patients were recruited. Formalin-fixed paraffin-embedded (FFPE) tumor tissue block and clinicopathological data were collected. Following tissue micro array (TMA) construction, 10 patients were excluded due to the lack of TMA cores, and 15 patients were excluded because the staining was unusable (folded core, or no tumor cells in the core). For 35 patients, HER2 status by IHC had not been performed, and for one patient ER and PR status had not been performed; hence, these patients were also excluded. In the end, 497 patients were available for the study.

For each patient, BC molecular subtypes were determined based on laboratory analysis and pathology reports. ER and PR status were collected from patient clinicopathological data. At CMS, the ASCO/CAP guidelines for ER and PR testing in BC are followed. HER2 status was analyzed in our laboratory using IHC (HerceptestTM). If the IHC status was equivocal, it was then evaluated by Fluorescence In Situ Hybridization (FISH), using the 2013 ASCO/CAP recommendations [29].

TMAs were constructed using 4 cores (0.6 mm of diameter) per patient, targeting the tumor, and confirmed by a breast pathologist. Uterus and kidney tissues, as well as different BC cell lines (SKBR3, MDA-MB-175, MDA-MB-231 and MCF-7), were used as controls. Four μm sections were cut for staining. Tumor cell morphology was verified by Hematoxylin and Eosin (H&E) staining.

All prevalence analyses were done using SAS (version 9.4).

### Cell lines selection

HER2 status verification was based on i) data from the literature, ii) American Type Culture Collection (ATCC), and iii) verification by IHC—Herceptest. pHER2^Y877^ status verification was based on i) data from the literature and ii) verification by IHC. We selected breast cancer cell lines according to HER2 and pHER2^Y877^ status. Twelve cell lines were analyzed in this study: BT-474, BT-549, BT-20, SKBR3, MDA-MB-453, MDA-MB-468, MDA-MB-231, MCF-7, SUM-149, SUM-159, ZR-75-1 (ATCC; Manassas, VA, USA) and JIMT-1 (kindly provided by Dr. Marcel B Bally, University of British Columbia, Vancouver). The cell lines were cultured as described below and included in paraffin blocks.

### Cell culture and paraffin fixation

BT-474 and BT-549 cells were cultured in RPMI-1640 (Wisent, 350-000-CL) media supplemented with 10% fetal bovine serum (FBS) (Wisent, 080–150), 1% penicillin-streptomycin (Penstrep) (Wisent, 450-200-EL), 10μg/ml insulin (Wisent 521–016). MDA-MB-468, MDA-MB-231, MDA-MB-453, SUM-149 and SUM-159 cells were maintained in RPMI-1640 media supplemented with 10% FBS and 1% penstrep. BT-20 cells were maintained in RPMI-1640 media supplemented with 10% FBS, 1% penstrep, 2mM L-glutamine (Wisent 609–065) and 1mM sodium pyruvate (Wisent 600–110). SKBR3 cells were maintained in McCoy’s 5a (Wisent, 217-010-XK) media supplemented with 10% FBS and 1% penstrep. MCF-7 cells were maintained in phenol red-free DMEM F12 (Wisent 319–080) supplemented with 5% FBS, 1% penstrep, 13.4 ml sodium bicarbonate 7.5% solution (Wisent 609–105), 7.5 ml HEPES 1M (Wisent 330–050) and 50μl estradiol 10-5M (E2) (Sigma E8875). ZR-75-1 cells were maintained in RPMI-1640 media supplemented with 10% FBS, 1% penstrep, 2mM L-glutamine, 7.5 ml HEPES, 13.4 ml sodium bicarbonate and 50μl E2. JIMT-1 cells were maintained with DMEM (Wisent, 319-005-CL) media supplemented with 10% FBS and 2mM L-glutamine. Cell lines were grown at 37°C with 5% CO2 and tested for mycoplasma infections (Lonza, MycoAlert PLUS). Cells were cultured to obtain 60–80 million cells, pelleted and suspended in formalin + 3% agar and FFPE.

### Immunohistochemistry of HER2 and pHER2^Y877^

HER2 protein expression was measured by IHC with the DAKO Herceptest^TM^ using the Herceptest^TM^ for automated link platform (SK001, Dako). Briefly, antigen retrieval was performed in PT Link (Dako, PT101) at 97°C for 40 min. Slides were then incubated with primary antibody (rabbit polyclonal antibody, A0485, Dako) for 30 min, followed by HRP-labeled polymer (SK001, Dako) for 30 min. Slides were developed with 3,3′-diaminobenzidine and counterstained with Mayers hematoxylin (SK308, Dako).

The pHER2^Y877^ status was determined using an antibody directed against phosphotyrosine 877 by IHC. Slides were deparaffinized in toluene then rehydrated in ethanol. Tris-EGTA buffer (pH9) was used for antigen retrieval (30 min, 95.6°C) in a water bath. Endogenous peroxidase and non-specific binding were blocked respectively with 0.3% H_2_O_2_ (diluted in methanol) and Super Block (IDetect). Slides were then incubated with primary antibody (Abcam, ab108371, rabbit monoclonal [EP2324Y] to ErbB2 phospho Y877) overnight at 4°C in a wet chamber, and then with the secondary antibody (Advance HRP Link, Dako) for 30 min. Slides were developed with 3,3′-diaminobenzidine and counterstained with Mayers hematoxylin. All washes were performed with 1X TBS.

Since HER2 and pHER2^Y877^ staining was similar, the 2013 ASCO/CAP guidelines were applied for pHER2^Y877^ as well (S1 Fig) [29]. For HER2 a score of 2+ by IHC corresponds to an equivocal HER2 status. When such a score is obtained, HER2 gene amplification using FISH can be performed to discriminate whether HER2 status is positive or negative [34]. However, it is not possible to perform such a cross-examination for pHER2^Y877^ expression, as gene amplification can not reflect HER2 phosphorylation status. Hence, we established that a score of 2+ in pHER2^Y877^ IHC staining would be considered positive. The pHER2^Y877^ staining was evaluated independently by a second person to assess reproducibility (n = 96, r = 0.82).

### Proliferation assay

Seven cell lines were selected according to their HER2/pHER2^Y877^ combination status. Cells were plated on 96-well plates in triplicate (10,000 cells per well). After 24 hours of incubation, trastuzumab (Roche-Genentech) was diluted in miliQ water (1 mg/ml) and added to cells at increasing concentrations (0, 4, 10, 20, 40, 60, 80, 100, 110, 120, 130, 140 and 150μg/ml). After 5 days of incubation, alamarBlue was added (10μl of alamarBlue for 100μl of media). Viability rate was determined by fluorescence. Percent viability was calculated for the treated cells compared to the untreated cells. All experiments were done in triplicate and mean ± SD of triplicates was calculated and plotted for each drug concentration.

All figures were generated using Prism/Graphpad (version 5). A two-way ANOVA test with an interaction term was used to examine differences between cell lines response to trastuzumab using the SAS software (version 9.4).

## Results

### Structure of HER2

**Fig 1:**
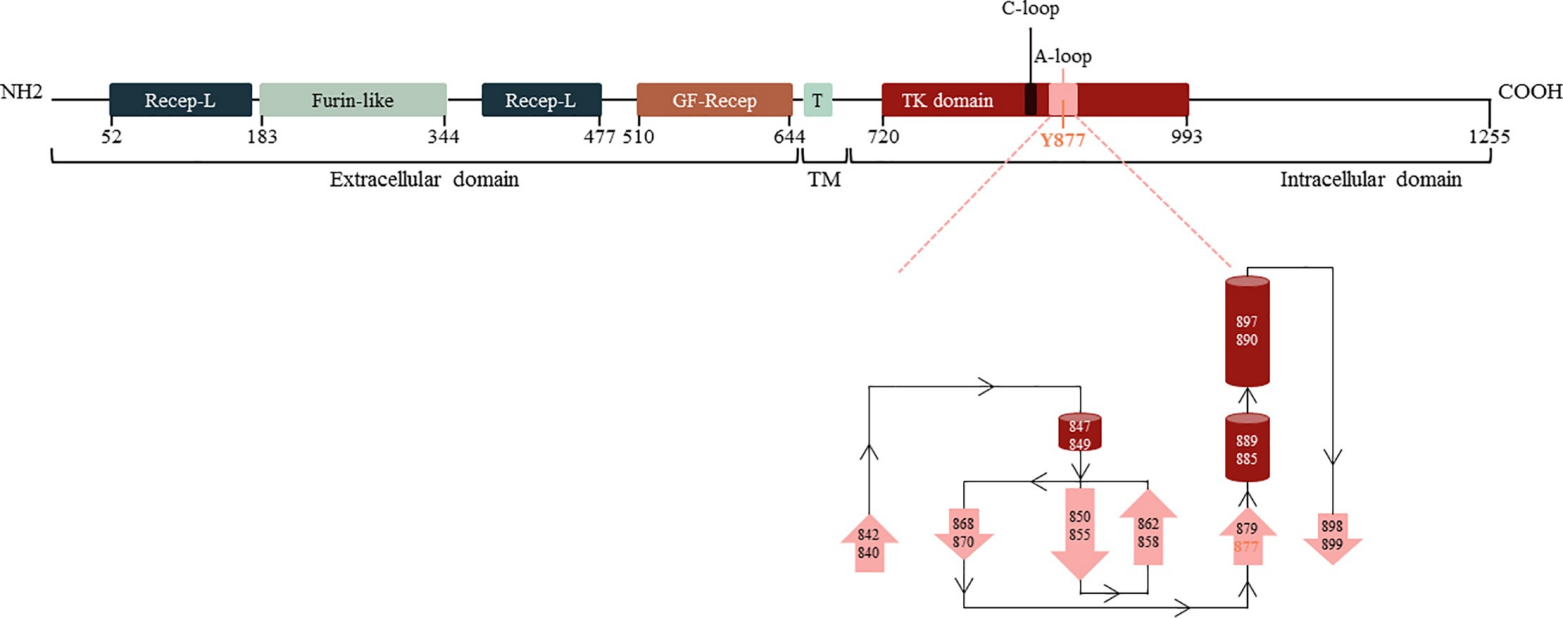
HER2 domains structure and activation loop. Y877 is localized in the activation loop of HER2 tyrosine kinase domain. Recep-L: Receptor-L-domain. Furin-like: Furin-like cysteine rich region domain. GF-Recep: Growth factor receptor domain IV. T and TM: Transmembrane domain. TK domain: Tyrosine-kinase domain. C-loop: Catalytic loop. A-loop: Activation loop. Arrows: beta sheets. Cylinders: alpha helix.

As shown in Fig 1, Y877 is localized in the intracellular domain, specifically in the tyrosine kinase activation loop. The activation loop is composed of five beta sheets and three alpha helixes, from R840 to F899. The Y877 is localized at the end of the activation loop. This region is critical for HER2 autophosphorylation and for the kinase activity regulation of the receptor [35].

### Prevalence of pHER2^Y877^

To evaluate pHER2^Y877^ clinical relevance, pHER2^Y877^ distribution was established in the BC population, as measured by the HER2^Y877^ prevalence in the BC patient cohort. As shown in Table 1, the cohort is composed of 497 BC patients, representative of the distribution of molecular BC subtypes, with 79.5% Luminal A, 9.3% Luminal B, 3.4% HER2 and 7.9% TNBC.

**Table 1:** Cohort description.

Molecular subtypes	n = 497 (%)
**Luminal A** (ER and/or PR+, HER2-)	395 (79.5)
**Luminal B\** (ER and/or PR+, HER2+)	46 (9.3)
**HER2** (ER-, PR-, HER2+)	17 (3.4)
**TNBC** (ER-, PR-, HER2-)	39 (7.9)

Breast cancer patients cohort, recruited between 2011 and 2012 at Centre des Maladies du Sein de Québec. Luminal A (ER+ and/or PR+, HER2-), Luminal B (ER+ and/or PR+, HER2+), HER2 (ER-, PR-, HER2+), and TNBC Triple Negative Breast Cancer (ER-, PR-, HER2-).

**Fig 2:**
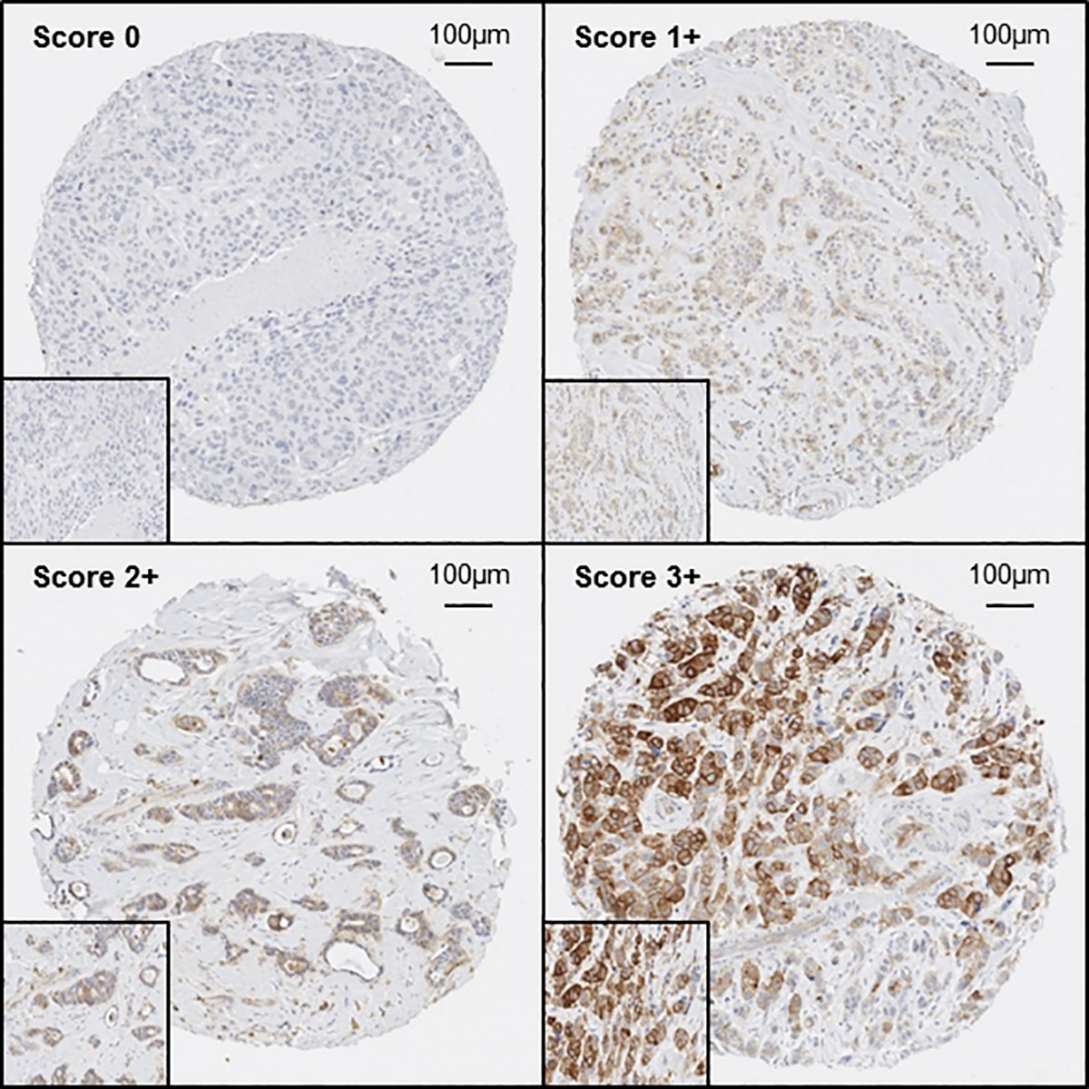
pHER2Y877 staining. The staining obtained is similar to HER2 with the four scores from the 2013 ASCO/CAP scoring guidelines. 0 = No staining is observed, or membrane staining is observed in less than 10% of tumor cells. 1+ = A faint/barely perceptible incomplete membrane staining is detected in more than 10% of tumor cells. 2+ = A weak to moderate complete membrane staining is observed in more than 10% of tumor cells. 3+ = A strong complete membrane staining is observed in more than 10% of tumor cells. The scoring defines a HER2 status; HER2 status is considered negative for scores of 0 and 1+, equivocal for a score of 2+ and positive for a score of 3+.

As depicted in Fig 2, pHER2^Y877^ expression was scored as 0 (no staining is observed or membrane staining is observed in <10% of the tumor cells), 1+ (a faint perceptible membrane staining is detected in >10% of tumor cells with incomplete membrane staining), 2+ (a weak to moderate complete membrane staining is observed in >10% of tumor cells) and 3+ (a strong complete membrane staining is observed in >10% of tumor cells), as described in the scoring guidelines of the 2013 ASCO/CAP.

**Fig 3:**
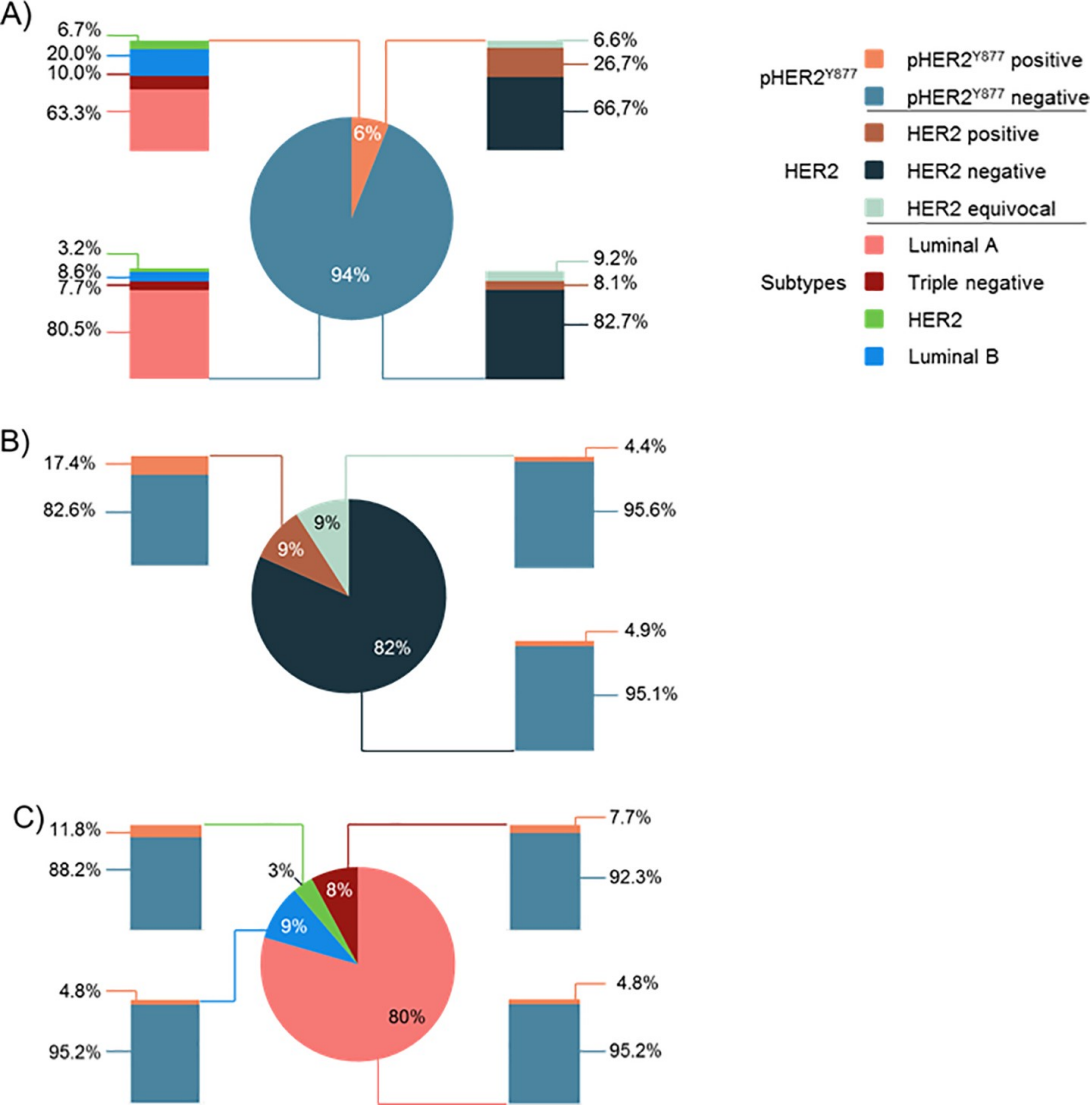
pHER2^Y877^ distribution. pHER2^Y877^ status was evaluated using the 2013 ASCO/CAP scoring guidelines after staining by IHC with anti-pHER2^Y877^ antibody. Score 2+ were considered as positive. HER2 status was evaluated by IHC (HercepTestTM kit) according to the 2013 ASCO/CAP scoring. Molecular subtypes were identified using the ER and PR status evaluates by IHC and the global HER2 status (IHC + FISH status). A) pHER2^Y877^ prevalence in the cohort. B) pHER2^Y877^ distribution according to HER2 status (defined by IHC). C) pHER2^Y877^ distribution according to molecular subtypes.

The pHER2^Y877^ prevalence was first established in our cohort of BC patients. As mentioned in Fig 3A, 6.0% of patients from our cohort overexpressed pHER2^Y877^. Among them, the distribution of HER2 status and molecular subtypes were representative of what is observed in the BC patient population at large (HER2 status: 6.6% equivocal, 26.7% positive and 66.7% negative; molecular subtypes: 6.7% HER2, 20.0% Luminal B, 10.0% TNBC and 63.3% Luminal A) [36]. This indicates that pHER2^Y877^ is well distributed among BC molecular subtypes and HER2 status, and is not specific to a subgroup in particular.

We then evaluated the distribution of pHER2^Y877^ staining according to HER2 status and molecular BC subtypes. HER2-negative BC represented 82% of the cohort; among them, 4.9% were pHER2^Y877^+. HER2+ and HER2-equivocal represented both 9% of the cohort; among them, 17.4% and 4.4% were pHER2^Y877^+, respectively (Fig 3B). Luminal A represented 80% of the cohort, and among this group 4.8% were pHER2^Y877^+. TNBC represented 8% of the cohort, and among them 7.7% were pHER2^Y877^+. Among HER2 (3%), 11.8% were pHER2^Y877^+. Finally among Luminal B (9%), 4.8% were pHER2^Y877^+ (Fig 3C).

As shown in Table 2, according to the Public Health Agency of Canada or the U.S Breast Cancer Statistics and the observed pHER2^Y877^ prevalence in our study, pHER2^Y877^+ overexpression could represent nearly 1,614 BC patients diagnosed in 2019 in Canada and 16,116 BC patients in the USA. The HER2−/pHER2^Y877^+ subgroup could represent 1,080 Canadian BC patients diagnosed in 2019 and 10,792 BC patients in the USA. As for TNBC patients who overexpress pHER2^Y877^_,_ they could represent 165 patients diagnosed in 2019 in Canada and 1655 BC patients in the USA.

**Table 2:** pHER2_Y877_ distribution according to estimation from Public Health Agency of Canada (PHAC) and U.S Breast Cancer Statistics estimation.

	BC	HER2-	TNBC
**N**	26,900/268,600	22,058/220,252	2152/21,488
**pHER2^Y877+^**	1614/16,116	1080/10,792	165/1655

Representation of pHER2_Y877_ prevalence according to our study and the PHAC or the U.S Breast Cancer Statistics.

BC: all BC patients diagnosed in 2019

HER2-: BC patients with a HER2-negative tumor

TNBC: Triple negative breast cancer

N: cases diagnosed in 2019 (Canada/U.S.A)

pHER2_Y877_+: number of patients positives to pHER2_Y877_

https://www.canada.ca/en/public-health/services/chronic-diseases/cancer/breast-cancer.html

https://www.breastcancer.org/symptoms/understand_bc/statistics

As a complement, FISH was also used to determine the HER2 status. The global HER2 status that combines scores obtained by both IHC and FISH was also evaluated. The results obtained are similar to those obtained by IHC (S2 and S3 Figs). However, for comparison purposes, only results obtained using IHC are shown because the same guidelines for staining evaluation were used.

### Influence of pHER2^Y877^on trastuzumab sensitivity

**Fig 4:**
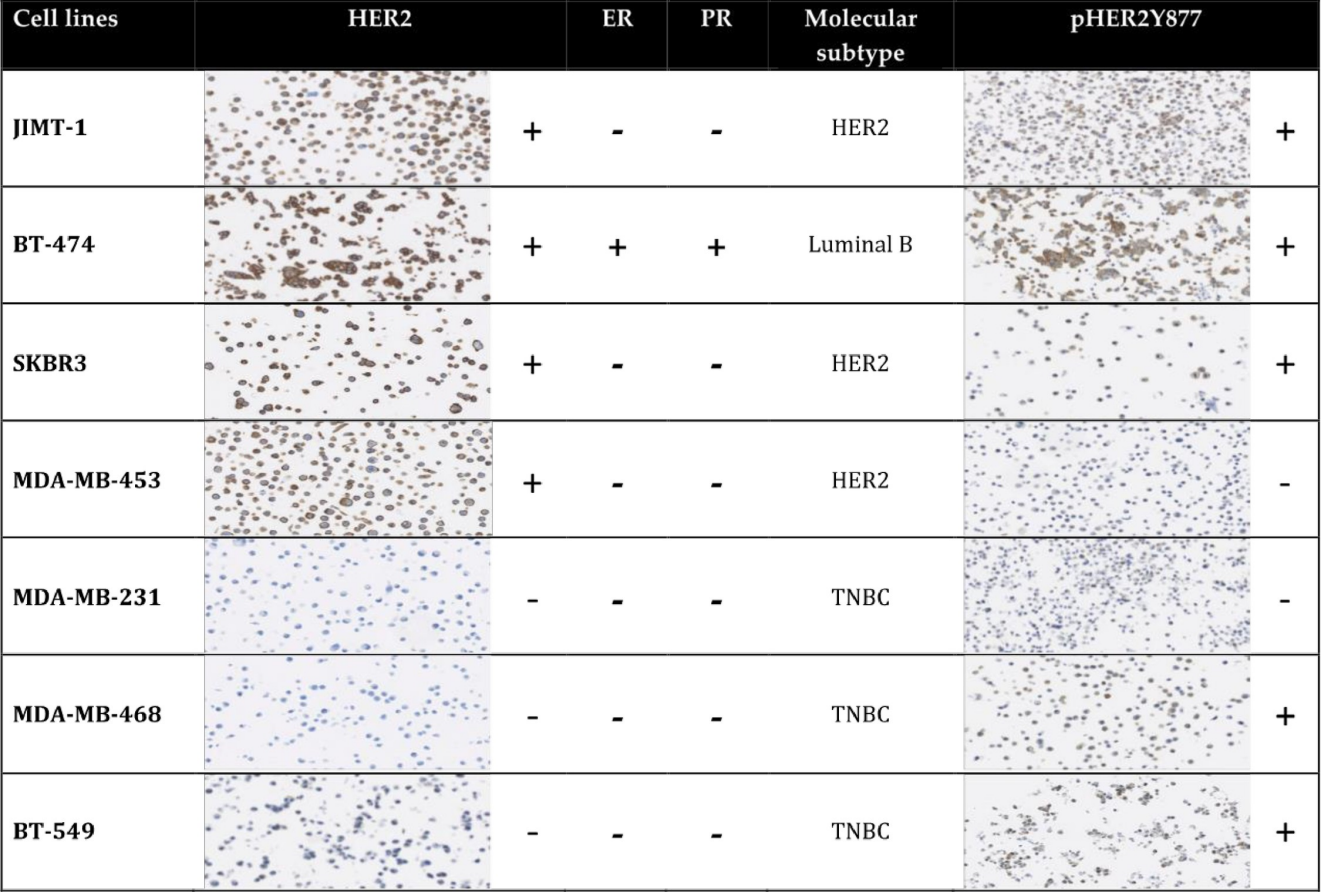
Brest cancer cell lines description. Breast cancer cell lines selected depending on their HER2 and pHER2^Y877^ status. HER2 status was obtained by using the Herceptest^TM^ kit–Dako and pHER2^Y877^ was using by IHC with a specific anti-pHER2^Y877^ antibody. ER (Estrogen Receptor) and PR (Progesterone Receptor) were confirmed by literature. HER2 (Human Epidermal Receptor 2), ER (Estrogen Receptor), PR (Progesteron Receptor), TNBC (Triple Negatif Breast Cancer).

HER2 and pHER2^Y877^ status were determined in a total of 12 selected BC cell lines. Seven BC cell lines were selected for further analysis based on their HER2 and pHER2^Y877^ status to have every status combination. As described in Fig 4, JIMT-1, BT-474, and SKBR3 are positive for both HER2 and pHER2^Y877^; MDA-MB-453 is positive for HER2 and negative for pHER2^Y87^; MDA-MB-231 is negative for both HER2 and pHER2^Y877^, while MDA-MB-468 and BT-549 are negative for HER2 and positive for pHER2^Y877^. Most of the additional HER2 negative BC cell lines were negative for both HER2 and pHER2^Y877^ (S4 Fig).

Breast cancer cell lines selected depending on their HER2 and pHER2^Y877^ status. HER2 status was obtained by using the Herceptest^TM^ kit–Dako and pHER2^Y877^ was using by IHC with a specific anti-pHER2^Y877^ antibody. ER (Estrogen Receptor) and PR (Progesterone Receptor) were confirmed by literature. HER2 (Human Epidermal Receptor 2), ER (Estrogen Receptor), PR (Progesteron Receptor), TNBC (Triple Negatif Breast Cancer).

**Fig 5:**
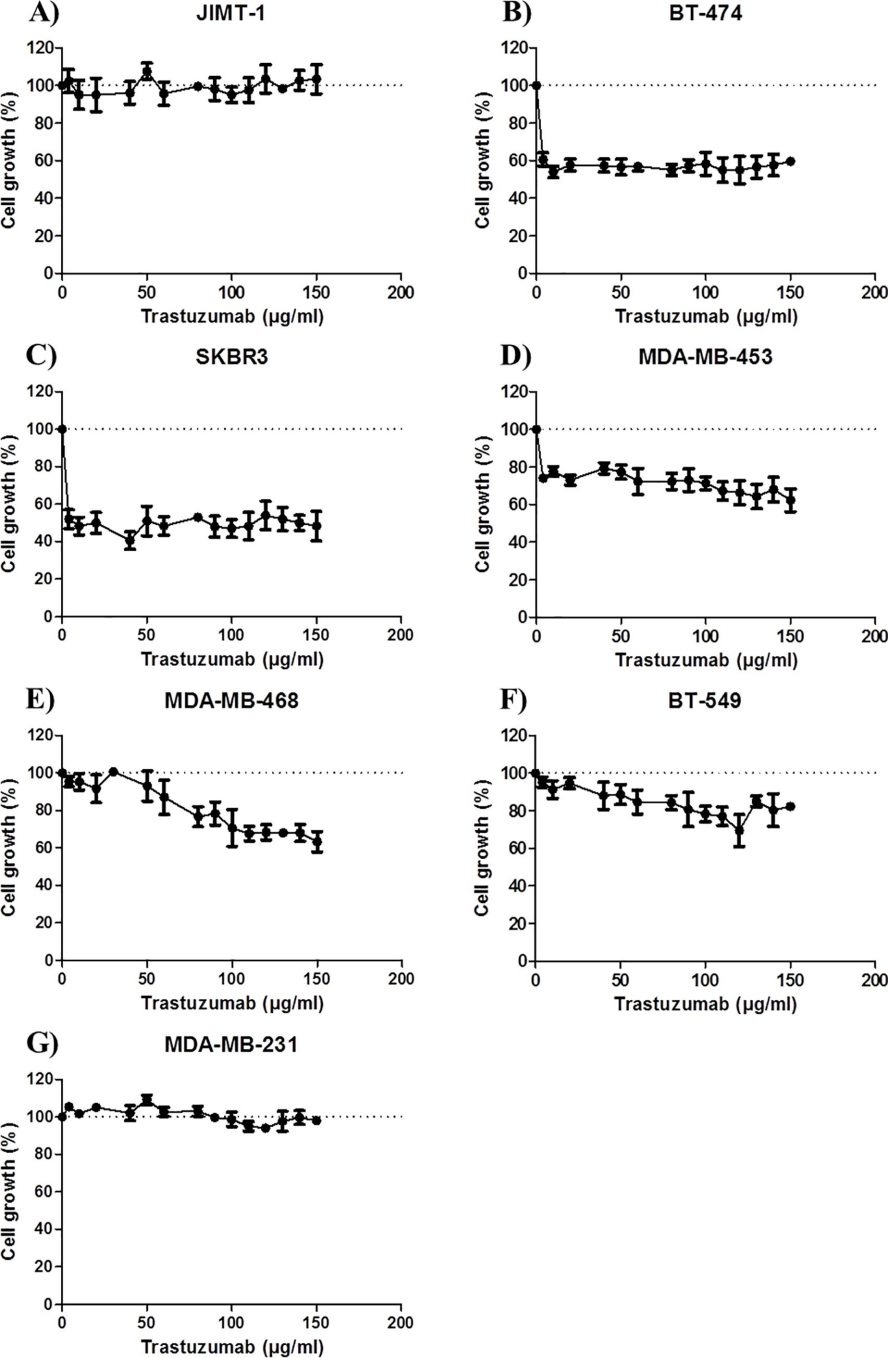
Breast cancer cell lines treated with trastuzumab in a dose-response manner. Proliferation assays have been performed for each cell line, treated with increasing doses of trastuzumab (0, 4, 10, 20, 40, 60, 80, 100, 110, 120, 130, 140 and 150μg/ml). The cell growth was calculated as the percentage of treated cells compared to untreated cells. All experiments were done in triplicates and means ± S-D were calculated and plotted for each drug concentration.

The JIMT-1 BC cell line was used as control as it is a known trastuzumab-resistant cell line [30]. This was confirmed in our proliferation assay; trastuzumab does not affect JIMT-1 proliferation even at high concentrations (Fig 5A). However, trastuzumab causes a similar decrease in proliferation in the two HER2+/pHER2^Y877^+ BC cell lines BT-474 and SKBR3 (p<0.0001 for both), as there is no difference between either the cell lines (p = 0.11) or the slopes (p = 0.99) (Fig 5B and 5C). This decrease occurs at a low trastuzumab concentration (4μg/ml) to reach approximately 57% and 50% cell viability for BT-474 and SKBR3, respectively, and then remains stable as trastuzumab concentrations increase.

As expected, trastuzumab is effective on MDA-MB-453, a HER2+/pHER2^Y877^− BC cell line, as a decrease in proliferation can be observed (Fig 5D). This decrease, however, is less important than that for BT-474 and SKBR3 as it reaches 72% cell viability. In fact, there is a difference between MDA-MB-453 and BT-474 or SKBR3 (p<0.0001 for both), but the slopes are similar (p = 0.77 for BT-474 and p = 0.35 for SKBR3) (S5A Fig).

Hence, trastuzumab acts similarly on MDA-MB-453 (HER2+/pHER2^Y877^−) and the HER2+/pHER2^Y877^+ cell lines but the decrease of proliferation is less important in MDA-MB-453 (HER2+/pHER2^Y877^−), indicating an added value of a phosphorylated Y877 in response to trastuzumab.

As anticipated, trastuzumab does not affect the proliferation of the TNBC cell line MDA-MB-231, which is HER2−/pHER2^Y877^−, even at high trastuzumab concentrations (Fig 5G). However, trastuzumab does have an effect on the TNBC cell line MDA-MB-468 proliferation, which is phosphorylated at Y877 of HER2 (Fig 5E). Proliferation assays show that MDA-MB-468 is significantly different from MDA-MB-231 and JIMT-1 either between cell lines (p<0.0001 for both), or the slopes (p = 0.002 for both). The TNBC cell line MDA-MB-468 (HER2−/pHER2^Y877^+) is sensitive to trastuzumab, but at higher doses (S5B Fig). As shown in S5 Fig, the decrease in proliferation starts at 50μg/ml to reach 67% cell viability. This decrease seems to be close to that found for MDA-MB-453. Overall, there is a difference between MDA-MB-468 and MDA-MB-453 in response to trastuzumab (p<0.0001), but the slopes are similar (p = 0.43). If we apply a trastuzumab cut-off of 90μg/ml, MDA-MB-468 proliferation seems to reach the one of MDA-MB-453 at this concentration, as they display the same response to trastuzumab (p = 0.43 for the difference between cell lines, and p = 0.99 for difference between the slopes) (S5C Fig).

However, trastuzumab is less efficient on BT-549 (HER2−/pHER2^Y877^+) proliferation (Fig 5F). Even though there is a significant difference between BT-549 and MDA-MB-231 or JIMT-1 overall in their response to trastuzumab (p<0.0001 for both), there is no difference in the concentrations (p ≥ 0.21 for both). This means that the decrease in proliferation driven by trastuzumab is not as pronounced for this particular cell line, as percent viability overlaps with MDA-MB-231 and JIMT-1 (S5B Fig).

Taken together, these results suggest that the decrease in proliferation induced by trastuzumab observed in the TNBC cell line MDA-MB-468 (HER2−/pHER2^Y877^+) is related to pHER2^Y877^ overexpression.

## Discussion

This is the first time that pHER2^Y877^ prevalence is established in a large cohort of BC patients. Most importantly, consecutive recruitment of patients was performed to provide a realistic view of BC patients visiting the Centre des Maladies du Sein in one year. This also limits the possibility of selection bias.

**Fig 6:**
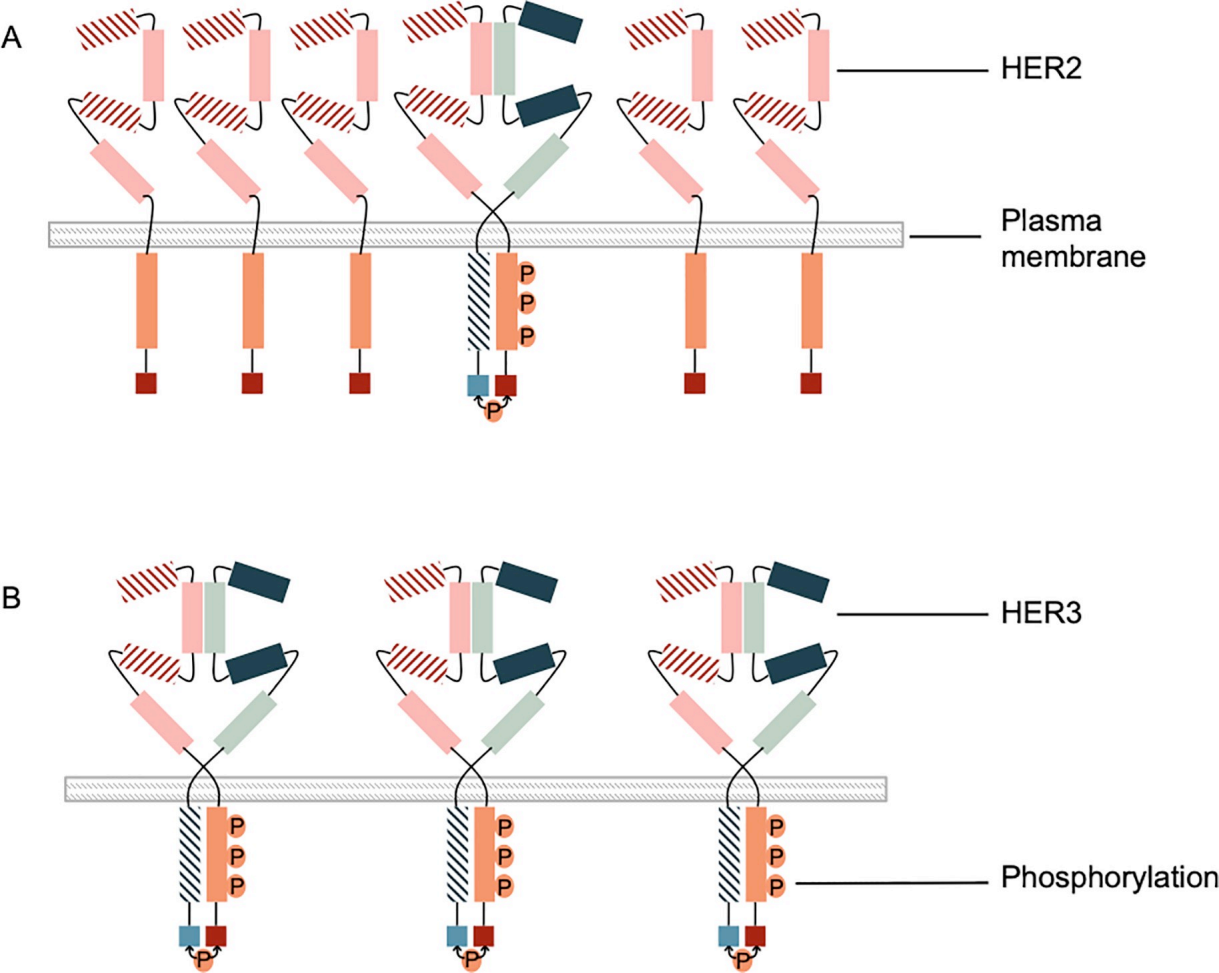
Overexpresion versus overphosphosrylation of HER2. A. Overexpression of HER2 with non-overphosphorylation of HER2 (HER2+; pHER2-). B. Non-overexpression of HER2 with overphosphorylation of HER2 (HER2-; pHER2+). In this study we show that BT-474 and SKBR3, which are HER2+/pHER2^Y877^+ have a better response to trastuzumab than MDA-MB-453, which is also HER2+ but not phosphorylated at Y877 (HER2+/pHER2^Y877^−). This is concordant with studies reporting that HER2 phosphorylation leads to a better response to trastuzumab in HER2-positive BC tumors [35–38]. As shown in a study by Giuliani *et al*., among HER2+ BC patients treated with trastuzumab, 89% with pHER2Y^1248^+ showed a positive response while only 49% of pHERY^1248^− presented a positive response [39]. These results suggest that the combination of HER2+/pHER^Y877^+ could indeed predict a better response to trastuzumab. However, our study is the first to examine HER2 phosphorylation at position Y877 in HER2-negative BC cell lines with regard to trastuzumab treatment. We demonstrated here that the decrease in proliferation in HER2-negative BC cell lines is Y877-phosphorylation-specific, as the TNBC cell line MDA-MB-468, which is HER2−/pHER2^Y877^+, displays sensitivity to trastuzumab. Studies have reported that Y877 phosphorylation is a marker of HER2 activation. This again is in agreement with our results showing that HER2 over-activation by over-phosphorylation at Y877 could be an additional biomarker in BC diagnosis.

Studies have evaluated the pHER2 prevalence at Y1248 and Y1221/1222 in BC patients cohorts, and among HER2− BC patients (IHC 0, 1+ or 2+). The prevalence of pHER2 at these positions was 0–38% and 10–15%, respectively [21–26]. In the present study, 82,6% of the HER2+ BC patients were pHER2^Y877^−. This is consistent with Ramic *et al*. where 55,8% of HER2+ BC patients were pHER2^Y1248^− [36]. In another study, Frogne *et al*. evaluated the pHER2^Y1221/1222^ prevalence in a cohort of 264 BC patients. They have shown that out of 242 HER2− BC patients, 9% were pHER2^Y1221/1222^+ (23 BC patients with strong pHER2^Y1221/1222^ staining) [25]. This is consistent with our findings that 6% of HER2− BC patients were pHER2^Y877^+ in our 497 BC patients cohort. Taken together and as pictured in Fig 5, these results show that even if HER2 is overexpressed, it is not necessarily over-phosphorylated, if the majority of HER2 is not dimerized and activated. On the other hand, even if HER2 is not overexpressed, if the majority is dimerized and activated it leads to an over-phosphorylation of HER2 (Fig 6).

However, trastuzumab sensitivity differs slightly for the TNBC cell line BT-549, which is also HER2−/pHER2^Y877^+. Although a decrease in proliferation is observed, it is not significantly different from MDA-MD-231 (HER2+/pHER2^Y877^−). This could also be the reflection of inter-individual response to trastuzumab treatment, similar to what is observed in BC patients. Worth mentioning is that TNBC can be classified into seven subtypes, such as Basal-like 1 (BL1), Basal-like 2 (BL2), Immunomodulatory (IM), Mesenchymal (M), Mesenchymal stem-like (MSL), Luminal androgen receptor (LAR) and Unstable (UNS). It has been shown that TNBC subtypes have different therapeutic responses [40]. Masuda *et al*. have reported that BL1 subtype has the highest pathologic complete response rate to neoadjuvant chemotherapy compared to the other TNBC subtypes [41]. MDA-MB-468 is of the BL1 subtype while BT-549 is of the M subtype [42]. This difference in subtype classification between those two TNBC cell lines could explain why BT-549 is less sensitive to trastuzumab than MDA-MB-468.

The BC cell lines included in this study were selected based on their HER2 and pHER2^Y877^ status. However, these cell lines also have other molecular characteristics. Multiple cell cycle proteins (RB1, p53, BRCA1 and BRCA2) and survival signaling pathways (PTEN and PIK3CA) are involved in BC and can affect the response to treatment [43,44]. Only MDA-MB-468 and BT-549 (HER2−/pHER2^Y877^+) have a loss of RB1 and PTEN compared with the other cell lines, but they are still sensitive to trastuzumab. MDA-MB-231 (HER2−/pHER2^Y877^−) has the same molecular characteristics for RB1, p53, PTEN, PI3KCA, BRCA1 and BRCA2 as SKBR3 and MDA-MB-453, which are both sensitive to trastuzumab (S1 Table). Hence, it seems that these proteins are not involved in the response to the trastuzumab.

We used trastuzumab concentrations ranging from 4μg/ml to 150μg/ml; these are commonly used in the literature [45,46]. However, it is difficult to evaluate the corresponding concentration in patients. To date, adjuvant trastuzumab is administered at an initial dose of 4mg/kg, and then at 2mg/kg weekly for 12 weeks, and finally at 6mg/kg every three weeks for 52 weeks. There is no official conversion between what is used in *in vitro* assays and what is currently given to BC patients. The decrease in proliferation in HER2−/pHER2^Y877^+ BC cell lines is observed at a trastuzumab concentration of 50μg/ml, while it appears at 4μg/ml for HER2+ BC cell lines. Therefore, HER2−/pHER2^Y877^+ BC patients would in theory require higher trastuzumab doses compared with HER2+ BC patients, while HER2+/pHER2^Y877^+ BC patients could receive a lesser dose.

Several preclinical studies have confirmed the biological importance of pHER2 as a mediator of cancer cell migration and proliferation. For example, in BC cell lines, pHER2 was associated to the activation of Src pathway, which regulates cell growth and proliferation. Moreover, inhibitors that specifically blocked pHER2 suppressed cancer cell migration *in vitro* [47,48]. In addition, inhibition of pHER2 led to decreased cell proliferation and increased cell death in three different BC cell lines [49]. Interestingly, one of those BC cell lines expressed low levels of HER2 [49]. A study from Wulfkuhle *et al*. has shown that TNBC patients receiving benefit from Neratinib, a tyrosine-kinase inhibitor used as an adjuvant therapy in BC, are pHER2^Y1248+^ [50]. This result is consistent with our findings that HER2−/pHER2^Y877+^ BC patients could be sensitive to trastuzumab. It also supports the fact that HER2 phosphorylation leading to its activation is important in HER2− BC sensitivity to HER2-targeted therapies.

Our study highlighted that pHER2^Y877^overexpression occurs in 6% of BC patients and renders HER2-negative BC cell lines sensitive to trastuzumab. Our results also suggest that there would be a benefit of adding the anti-pHER2^Y877^ antibody along with HercepTest at the time of diagnosis to target HER2−/pHER2^Y877^+ BC patients. These patients could then be treated with adequate doses of trastuzumab.

Our study could have important clinical impacts. According to PHAC, 26,900 BC patients were diagnosed in Canada in 2019. Considering the observed pHER2^Y877^+ prevalence among HER2 negative BC patients in our study, we estimate that 1,080 BC patients were HER2−/pHER2^Y877^+ in Canada in 2019 and could have benefited from trastuzumab. This finding has huge impact for BC patients and their families. Among these BC patients, some have tumors of the Luminal A subtype, which can be treated by hormone therapy but could also be treated with trastuzumab. Most importantly, there is, as yet, no personalized therapy to treat TNBC [51]. This study implies that more than 160 TNBC patients could be eligible to receive trastuzumab treatment in Canada alone. This is an immense step toward precision medicine for these women.

These results need however to be confirmed in a bigger cohort, as well as with *in vivo* experiments, such as xenografts and/or PDX models to test the effect of trastuzumab depending on HER2 phosphorylation. However, our study is a promising first step in personalized treatment research for HER2-negative BC patients, for which a tailored therapy is still an unmet need.

## Supporting information

S1 Fig Guidelines for HER2 scoring. Adapted from the HercepTest^TM^ interpretation manual (Dako, Agilent). Guidelines are enacted by the American Society of Clinical Oncology/College of American Pathologist. 0 = No staining is observed, or membrane staining is observed in less than 10% of tumor cells. 1+ = A faint/barely perceptible incomplete membrane staining is detected in more than 10% of tumor cells. 2+ = A weak to moderate complete membrane staining is observed in more than 10% of tumor cells. 3+ = A strong complete membrane staining is observed in more than 10% of tumor cells. The scoring defines a HER2 status is considered negative for scores 0 and 1+, equivocal for a score of 2+ and positive for score 3+.
(TIF)

S2 Fig pHER2^Y877^ distribution–HER2 status by FISH. pHER2^Y877^ status was evaluated using the 2013 ASCO/CAP scoring guidelines after staining by IHC with anti-pHER2^Y877^ antibody. A score of 2+ pHER2^Y877^ staining were considered positive. HER2 status was evaluated using the 2013 ASCO/CAP guidelines after staining by FISH (Fluorescence In Situ Hybridization). Molecular subtypes were identified using the ER and PR status evaluated by IHC and the global HER2 status (IHC + FISH status). A) pHER2^Y877^ prevalence in the cohort. B) pHER2^Y877^ distribution according to HER2 status, defined by FISH.
(TIF)

S3 Fig pHER2^Y877^ distribution—global HER2 status. pHER2^Y877^ status was evaluated using the 2013 ASCO/CAP scoring guidelines after staining by IHC with anti-pHER2^Y877^ antibody. A score of 2+ pHER2^Y877^ staining was considered positive. HER2 status was evaluated using the 2013 ASCO/CAP guidelines after staining by FISH and IHC. Molecular subtypes were identified using the ER and PR status evaluated by IHC and the global HER2 status (IHC + FISH status). A) pHER2^Y877^ prevalence in the cohort. B) pHER2^Y877^ distribution according to HER2 status, defined by IHC+FISH.
(TIF)

S4 Fig HER2 and pHER2Y877 status in additional breast cancer cell lines. HER2 status was obtained using the HerceptestTM kit–Dako (left column), while pHER2^Y877^ was performed by IHC with a specific anti-pHER2^Y877^ antibody (right column). BT-20: HER2-negative; pHER2^Y877^-positive. MCF7: HER2-negative; pHER2^Y877^-negative. SUM-149: HER2-negative; pHER2^Y877^-negative. SUM-159: HER2-negative; pHER2^Y877^-negative. ZR-75-1: HER2-equivocal; pHER2^Y877^-negative.
(TIF)

S5 Fig Comparison of proliferation assay of breast cancer cell lines treated with trastuzumab. Each cell line was treated with increasing doses of trastuzumab (0, 4, 10, 20, 40, 60, 80, 100, 110, 120, 130, 140 and 150μg/ml). The cell growth was calculated as the percentage of treated cells compared to untreated cells. All experiments were done in triplicates and means ± SD were calculated and plotted for each drug concentration. ANOVA has been done using SAS software. A) Comparison of BT-474 and SKBR3 to MDA-MB-453. B) Comparison of MDA-MB-468 and BT-549 to JIMT-1 and MDA-MB-231. C) Comparison of MDA-MB-468 and BT549 to MDA-MB-453. * p < 0.0001 between cell lines.
(TIF)

S1 Table Characeristics of breast cancer cell ines. Protein expression and gene mutation in breast cancer cell line according to their molecular subtype. +: protein expressed; -: protein not expressed. mut: gene mutated; wt: gene wild-type.
(TIFF)

## References

[1] Dai X, Xiang L, Li T, Bai Z. Cancer Hallmarks, Biomarkers and Breast Cancer Molecular Subtypes. J Cancer. 2016;7(10):1281–94. 10.7150/jca.13141 27390604 PMC4934037

[2] Fulford LG, Easton DF, Reis-Filho JS, Sofronis A, Gillett CE, Lakhani SR, et al Specific morphological features predictive for the basal phenotype in grade 3 invasive ductal carcinoma of breast: *Morphological features predictive for basal phenotype in grade 3 IDC of breast*. Histopathology. 2006 7;49(1):22–34. 10.1111/j.1365-2559.2006.02453.x 16842243

[3] Hudelist G, Köstler WJ, Attems J, Czerwenka K, Müller R, Manavi M, et al Her-2/neu-triggered intracellular tyrosine kinase activation: in vivo relevance of ligand-independent activation mechanisms and impact upon the efficacy of trastuzumab-based treatment. Br J Cancer. 2003 9 15;89(6):983–91. 10.1038/sj.bjc.6601160 12966413 PMC2376939

[4] Downward J, Yarden Y, Mayes E, Scrace G, Totty N, Stockwell P, et al Close similarity of epidermal growth factor receptor and v-erb-B oncogene protein sequences. Nature. 1984 2 9;307(5951):521–7. 10.1038/307521a0 6320011

[5] Kraus MH, Issing W, Miki T, Popescu NC, Aaronson SA. Isolation and characterization of ERBB3, a third member of the ERBB/epidermal growth factor receptor family: evidence for overexpression in a subset of human mammary tumors. Proc Natl Acad Sci USA. 1989 12;86(23):9193–7. 10.1073/pnas.86.23.9193 2687875 PMC298460

[6] Plowman GD, Culouscou JM, Whitney GS, Green JM, Carlton GW, Foy L, et al Ligand-specific activation of HER4/p180erbB4, a fourth member of the epidermal growth factor receptor family. Proc Natl Acad Sci USA. 1993 3 1;90(5):1746–50. 10.1073/pnas.90.5.1746 8383326 PMC45956

[7] Ullrich A, Schlessinger J. Signal transduction by receptors with tyrosine kinase activity. Cell. 1990 4 20;61(2):203–12. 10.1016/0092-8674(90)90801-k 2158859

[8] Jorissen RN, Walker F, Pouliot N, Garrett TPJ, Ward CW, Burgess AW. Epidermal growth factor receptor: mechanisms of activation and signalling. Exp Cell Res. 2003 3 10;284(1):31–53. 10.1016/s0014-4827(02)00098-8 12648464

[9] Roskoski R. The ErbB/HER receptor protein-tyrosine kinases and cancer. Biochem Biophys Res Commun. 2004 6 18;319(1):1–11. 10.1016/j.bbrc.2004.04.150 15158434

[10] Yarden Y, Sliwkowski MX. Untangling the ErbB signalling network. Nat Rev Mol Cell Biol. 2001 2;2(2):127–37. 10.1038/35052073 11252954

[11] Roskoski R. ErbB/HER protein-tyrosine kinases: Structures and small molecule inhibitors. Pharmacol Res. 2014 9;87:42–59. 10.1016/j.phrs.2014.06.001 24928736

[12] Slamon DJ, Clark GM, Wong SG, Levin WJ, Ullrich A, McGuire WL. Human breast cancer: correlation of relapse and survival with amplification of the HER-2/neu oncogene. Science. 1987 1 9;235(4785):177–82. 10.1126/science.3798106 3798106

[13] Shawver LK, Slamon D, Ullrich A. Smart drugs: tyrosine kinase inhibitors in cancer therapy. Cancer Cell. 2002 3;1(2):117–23. 10.1016/s1535-6108(02)00039-9 12086869

[14] Paik S, Kim C, Wolmark N. HER2 status and benefit from adjuvant trastuzumab in breast cancer. N Engl J Med. 2008 3 27;358(13):1409–11. 10.1056/NEJMc0801440 18367751

[15] Gyawali B, Niraula S. Duration of adjuvant trastuzumab in HER2 positive breast cancer: Overall and disease free survival results from meta-analyses of randomized controlled trials. Cancer Treatment Reviews. 2017 11;60:18–23. 10.1016/j.ctrv.2017.08.001 28863313

[16] Gianni L, Dafni U, Gelber RD, Azambuja E, Muehlbauer S, Goldhirsch A, et al Treatment with trastuzumab for 1 year after adjuvant chemotherapy in patients with HER2-positive early breast cancer: a 4-year follow-up of a randomised controlled trial. The Lancet Oncology. 2011 3;12(3):236–44. 10.1016/S1470-2045(11)70033-X 21354370

[17] Piccart-Gebhart MJ, Procter M, Leyland-Jones B, Goldhirsch A, Untch M, Smith I, et al Trastuzumab after adjuvant chemotherapy in HER2-positive breast cancer. N Engl J Med. 2005 10 20;353(16):1659–72. 10.1056/NEJMoa052306 16236737

[18] Ross JS, Slodkowska EA, Symmans WF, Pusztai L, Ravdin PM, Hortobagyi GN. The HER-2 receptor and breast cancer: ten years of targeted anti-HER-2 therapy and personalized medicine. Oncologist. 2009 4;14(4):320–68. 10.1634/theoncologist.2008-0230 19346299

[19] Weigelt B, Lo AT, Park CC, Gray JW, Bissell MJ. HER2 signaling pathway activation and response of breast cancer cells to HER2-targeting agents is dependent strongly on the 3D microenvironment. Breast Cancer Res Treat. 2010 7;122(1):35–43. 10.1007/s10549-009-0502-2 19701706 PMC2935800

[20] Olayioye MA. Intracellular signaling pathways of ErbB2/HER-2 and family members. Breast Cancer Res. 2001 12;3(6):385 10.1186/bcr327 11737890 PMC138705

[21] DiGiovanna MP, Stern DF, Edgerton SM, Whalen SG, Moore D, Thor AD. Relationship of Epidermal Growth Factor Receptor Expression to ErbB-2 Signaling Activity and Prognosis in Breast Cancer Patients. JCO. 2005 2 20;23(6):1152–60. 10.1200/JCO.2005.09.055 15718311

[22] Haas S, Gevensleben H, Rabstein S, Harth V, Pesch B, Brüning T, et al Expression of heregulin, phosphorylated HER-2, HER-3 and HER-4 in HER-2 negative breast cancers. Oncol Rep. 2009 2;21(2):299–304. 19148499

[23] Thor AD, Liu S, Edgerton S, Moore D, Kasowitz KM, Benz CC, et al Activation (Tyrosine Phosphorylation) of ErbB-2 (HER-2/neu): A Study of Incidence and Correlation With Outcome in Breast Cancer. JCO. 2000 9 18;18(18):3230–9. 10.1200/JCO.2000.18.18.3230 10986055

[24] Singer CF, Gschwantler-Kaulich D, Fink-Retter A, Pfeiler G, Walter I, Hudelist G, et al HER2 overexpression and activation, and tamoxifen efficacy in receptor-positive early breast cancer. J Cancer Res Clin Oncol. 2009 6;135(6):807–13. 10.1007/s00432-008-0516-x 19034514 PMC12160178

[25] Frogne T, Laenkholm A-V, Lyng MB, Henriksen KL, Lykkesfeldt AE. Determination of HER2 phosphorylation at tyrosine 1221/1222 improves prediction of poor survival for breast cancer patients with hormone receptor-positive tumors. Breast Cancer Res. 2009 6;11(1):R11 10.1186/bcr2230 19239686 PMC2687716

[26] Larsen MS, Bjerre K, Lykkesfeldt AE, Giobbie-Hurder A, Lænkholm A-V, Henriksen KL, et al Activated HER-receptors in predicting outcome of ER-positive breast cancer patients treated with adjuvant endocrine therapy. The Breast. 2012 10;21(5):662–8. 10.1016/j.breast.2012.07.005 22854050 PMC3438354

[27] Spears M, Pederson HC, Lyttle N, Gray C, Quintayo MA, Brogan L, et al Expression of activated type I receptor tyrosine kinases in early breast cancer. Breast Cancer Res Treat. 2012 7;134(2):701–8. 10.1007/s10549-012-2076-7 22562124

[28] Cicenas J, Urban P, Küng W, Vuaroqueaux V, Labuhn M, Wight E, et al Phosphorylation of tyrosine 1248-ERBB2 measured by chemiluminescence-linked immunoassay is an independent predictor of poor prognosis in primary breast cancer patients. European Journal of Cancer. 2006 3;42(5):636–45. 10.1016/j.ejca.2005.11.012 16414259

[29] Wolff AC, Hammond MEH, Hicks DG, Dowsett M, McShane LM, Allison KH, et al Recommendations for human epidermal growth factor receptor 2 testing in breast cancer: American Society of Clinical Oncology/College of American Pathologists clinical practice guideline update. J Clin Oncol. 2013 11 1;31(31):3997–4013. 10.1200/JCO.2013.50.9984 24101045

[30] Tanner M, Kapanen AI, Junttila T, Raheem O, Grenman S, Elo J, et al Characterization of a novel cell line established from a patient with Herceptin-resistant breast cancer. Mol Cancer Ther. 2004 12;3(12):1585–92. 15634652

[31] Telesco SE, Shih A, Liu Y, Radhakrishnan R. Investigating Molecular Mechanisms of Activation and Mutation of the HER2 Receptor Tyrosine Kinase through Computational Modeling and Simulation. Cancer Res J. 2011;4(4):1–35. 25346782 PMC4208668

[32] Hu Z, Wan X, Hao R, Zhang H, Li L, Li L, et al Phosphorylation of Mutationally Introduced Tyrosine in the Activation Loop of HER2 Confers Gain-of-Function Activity. Tagliabue E, editor. PLoS ONE. 2015 4 8;10(4):e0123623 10.1371/journal.pone.0123623 25853726 PMC4390223

[33] Furrer D, Jacob S, Caron C, Sanschagrin F, Provencher L, Diorio C. Concordance of HER2 Immunohistochemistry and Fluorescence In Situ Hybridization Using Tissue Microarray in Breast Cancer. Anticancer Res. 2017;37(6):3323–9. 10.21873/anticanres.11701 28551685

[34] Kawaguchi Y, Kono K, Mimura K, Mitsui F, Sugai H, Akaike H, et al Targeting EGFR and HER-2 with cetuximab- and trastuzumab-mediated immunotherapy in oesophageal squamous cell carcinoma. British Journal of Cancer. 2007 8;97(4):494–501. 10.1038/sj.bjc.6603885 17622245 PMC2360355

[35] Bose R, Molina H, Patterson AS, Bitok JK, Periaswamy B, Bader JS, et al Phosphoproteomic analysis of Her2/neu signaling and inhibition. Proceedings of the National Academy of Sciences. 2006 6 27;103(26):9773–8. 10.1073/pnas.0603948103 PMC1502529 16785428

[36] Ramić S, Asić K, Balja MP, Paić F, Benković V, Knežević F. Correlation of phosphorylated HER2 with clinicopathological characteristics and efficacy of trastuzumab treatment for breast cancer. Anticancer Res. 2013 6;33(6):2509–15. 23749902

[37] Ginestier C, Adélaïde J, Gonçalvès A, Repellini L, Sircoulomb F, Letessier A, et al ERBB2 phosphorylation and trastuzumab sensitivity of breast cancer cell lines. Oncogene. 2007 11 1;26(50):7163–9. 10.1038/sj.onc.1210528 17525746

[38] Dokmanovic M, Wu Y, Shen Y, Chen J, Hirsch DS, Wu WJ. Trastuzumab-induced recruitment of Csk-homologous kinase (CHK) to ErbB2 receptor is associated with ErbB2-Y1248 phosphorylation and ErbB2 degradation to mediate cell growth inhibition. Cancer Biology & Therapy. 2014 8;15(8):1029–41. 24835103 10.4161/cbt.29171 PMC4119070

[39] Giuliani R, Durbecq V, Di Leo A, Paesmans M, Larsimont D, Leroy J-Y, et al Phosphorylated HER-2 tyrosine kinase and Her-2/neu gene amplification as predictive factors of response to trastuzumab in patients with HER-2 overexpressing metastatic breast cancer (MBC). European Journal of Cancer. 2007 3;43(4):725–35. 10.1016/j.ejca.2006.11.019 17251007

[40] Kalimutho M, Parsons K, Mittal D, López JA, Srihari S, Khanna KK. Targeted Therapies for Triple-Negative Breast Cancer: Combating a Stubborn Disease. Trends in Pharmacological Sciences. 2015 12;36(12):822–46. 10.1016/j.tips.2015.08.009 26538316

[41] Masuda H, Baggerly KA, Wang Y, Zhang Y, Gonzalez-Angulo AM, Meric-Bernstam F, et al Differential Response to Neoadjuvant Chemotherapy Among 7 Triple-Negative Breast Cancer Molecular Subtypes. Clinical Cancer Research. 2013 10 1;19(19):5533–40. 10.1158/1078-0432.CCR-13-0799 23948975 PMC3813597

[42] Tseng L-M, Chiu J-H, Liu C-Y, Tsai Y-F, Wang Y-L, Yang C-W, et al A comparison of the molecular subtypes of triple-negative breast cancer among non-Asian and Taiwanese women. Breast Cancer Res Treat. 2017 6;163(2):241–54. 10.1007/s10549-017-4195-7 28299476 PMC5410215

[43] Landberg G, Roos G. The cell cycle in breast cancer. APMIS. 1997 7;105(7–12):575–89. 9298094 10.1111/j.1699-0463.1997.tb05056.x

[44] Stemke-Hale K, Gonzalez-Angulo AM, Lluch A, Neve RM, Kuo W-L, Davies M, et al An Integrative Genomic and Proteomic Analysis of PIK3CA, PTEN, and AKT Mutations in Breast Cancer. Cancer Research. 2008 8 1;68(15):6084–91. 10.1158/0008-5472.CAN-07-6854 18676830 PMC2680495

[45] Hudelist G, Köstler WJ, Czerwenka K, Kubista E, Attems J, Müller R, et al Her-2/neu and EGFR tyrosine kinase activation predict the efficacy of trastuzumab-based therapy in patients with metastatic breast cancer. International Journal of Cancer. 2006 3 1;118(5):1126–34. 10.1002/ijc.21492 16161043

[46] Demirci Alanyali S, Bozkurt E, Alanyali H, Karaca B, Uslu R. Radiosensitization of HER2-positive breast cancer cell lines with trastuzumab. JCO. 2013 5 20;31(15_suppl):e11501–e11501.

[47] Cabioglu N, Summy J, Miller C, Parikh NU, Sahin AA, Tuzlali S, et al CXCL-12/Stromal Cell–Derived Factor-1α Transactivates HER2-neu in Breast Cancer Cells by a Novel Pathway Involving Src Kinase Activation. Cancer Res. 2005 8 1;65(15):6493–7. 10.1158/0008-5472.CAN-04-1303 16061624

[48] Montgomery RB, Makary E, Schiffman K, Goodell V, Disis ML. Endogenous anti-HER2 antibodies block HER2 phosphorylation and signaling through extracellular signal-regulated kinase. Cancer Res. 2005 1 15;65(2):650–6. 15695410

[49] Pignatelli M, Cortés-Canteli M, Lai C, Santos A, Perez-Castillo A. The peroxisome proliferator-activated receptor gamma is an inhibitor of ErbBs activity in human breast cancer cells. J Cell Sci. 2001 11;114(Pt 22):4117–26. 11739643 10.1242/jcs.114.22.4117

[50] Wulfkuhle JD, Yau C, Wolf DM, Vis DJ, Gallagher RI, Brown-Swigart L, et al Evaluation of the HER/PI3K/AKT Family Signaling Network as a Predictive Biomarker of Pathologic Complete Response for Patients With Breast Cancer Treated With Neratinib in the I-SPY 2 TRIAL. JCO Precision Oncology. 2018 11;(2):1–20. 32914002 10.1200/PO.18.00024 PMC7446527

[51] Thakur V, Kutty RV. Recent advances in nanotheranostics for triple negative breast cancer treatment. J Exp Clin Cancer Res. 2019 12;38(1):430 10.1186/s13046-019-1443-1 31661003 PMC6819447

