# Correction: Trastuzumab effects depend on HER2 phosphorylation in HER2-negative breast cancer cell lines

**DOI:** 10.1371/journal.pone.0241089

**Published:** 2020-10-15

**Authors:** The PLOS ONE Staff

There is an error in the caption for Fig 6. The caption includes text that should be placed between the second and third paragraphs of the Discussion. The publisher apologizes for the error. Please see the complete, correct Fig 6 caption here.

**Fig 6:**
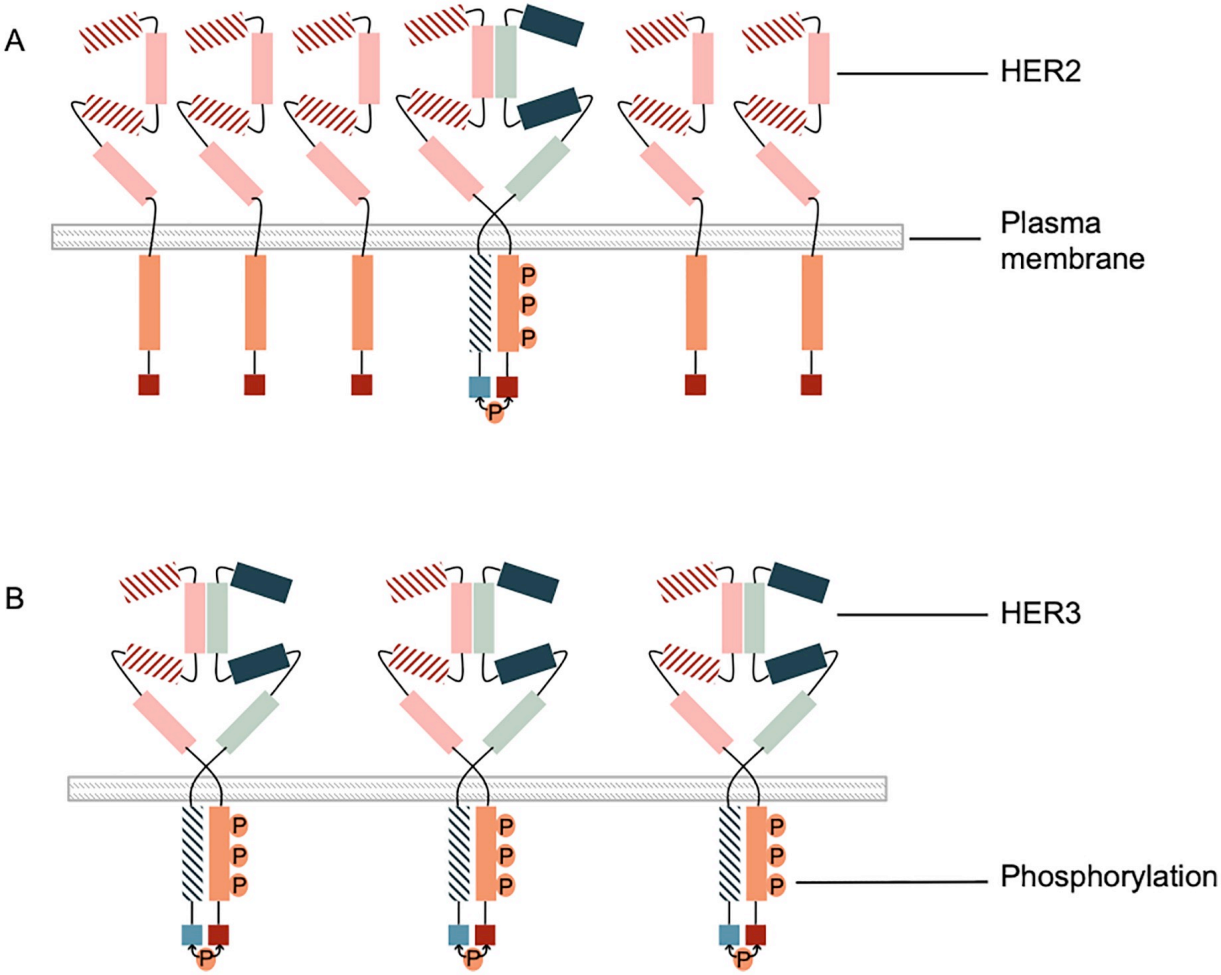
Overexpresion versus overphosphosrylation of HER2. A. Overexpression of HER2 with non-overphosphorylation of HER2 (HER2+; pHER2-). B. Non-overexpression of HER2 with overphosphorylation of HER2 (HER2-; pHER2+).

As a result, there is a paragraph missing between the second and third paragraphs of the Discussion. Please see the missing text here.

In this study we show that BT-474 and SKBR3, which are HER2+/pHER2^Y877^+ have a better response to trastuzumab than MDA-MB-453, which is also HER2+ but not phosphorylated at Y877 (HER2+/pHER2^Y877^−). This is concordant with studies reporting that HER2 phosphorylation leads to a better response to trastuzumab in HER2-positive BC tumors [35–38]. As shown in a study by Giuliani *et al*., among HER2+ BC patients treated with trastuzumab, 89% with pHER2Y^1248^+ showed a positive response while only 49% of pHERY^1248^− presented a positive response [39]. These results suggest that the combination of HER2+/pHER^Y877^+ could indeed predict a better response to trastuzumab. However, our study is the first to examine HER2 phosphorylation at position Y877 in HER2-negative BC cell lines with regard to trastuzumab treatment. We demonstrated here that the decrease in proliferation in HER2-negative BC cell lines is Y877-phosphorylation-specific, as the TNBC cell line MDA-MB-468, which is HER2−/pHER2^Y877^+, displays sensitivity to trastuzumab. Studies have reported that Y877 phosphorylation is a marker of HER2 activation. This again is in agreement with our results showing that HER2 over-activation by over-phosphorylation at Y877 could be an additional biomarker in BC diagnosis.
