# Supplementary figures and images for: Trastuzumab effects depend on HER2 phosphorylation in HER2-negative breast cancer cell lines
Source: PLoS One. 2020 Jun 25;15(6):e0234991. doi: 10.1371/journal.pone.0234991 (PMC7316326; doi:10.1371/journal.pone.0234991)

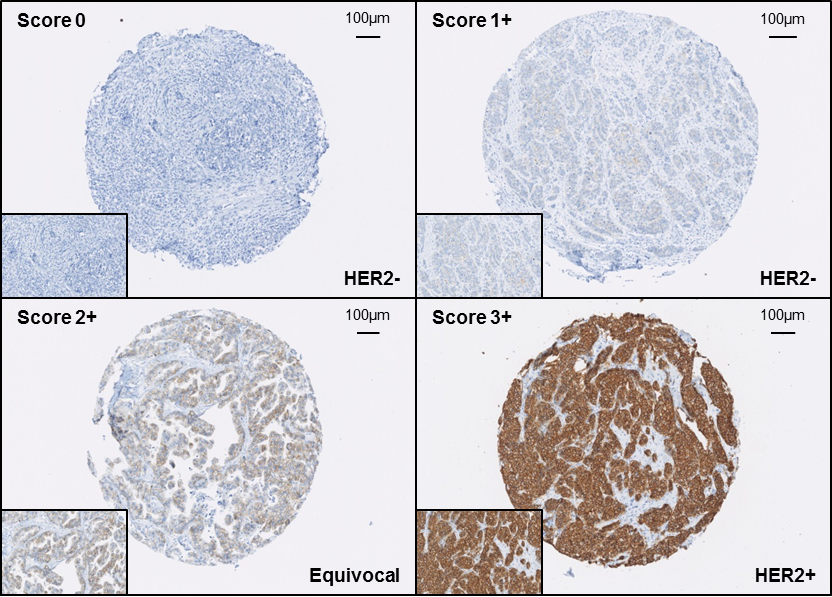

Supplement: S1 Fig — Adapted from the HercepTestTM interpretation manual (Dako, Agilent). Guidelines are enacted by the American Society of Clinical Oncology/College of American Pathologist. 0 = No staining is observed, or membrane staining is observed in less than 10% of tumor cells. 1+ = A faint/barely perceptible incomplete membrane staining is detected in more than 10% of tumor cells. 2+ = A weak to moderate complete membrane staining is observed in more than 10% of tumor cells. 3+ = A strong complete membrane staining is observed in more than 10% of tumor cells. The scoring defines a HER2 status is considered negative for scores 0 and 1+, equivocal for a score of 2+ and positive for score 3+. (TIF) [file pone.0234991.s001.tif]

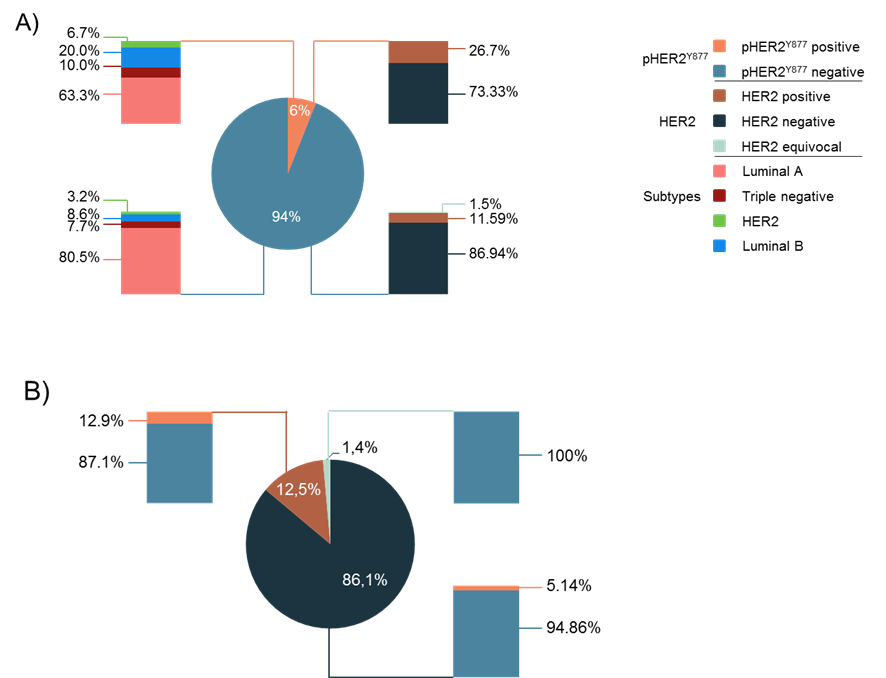

Supplement: S2 Fig — pHER2Y877 status was evaluated using the 2013 ASCO/CAP scoring guidelines after staining by IHC with anti-pHER2Y877 antibody. A score of 2+ pHER2Y877 staining were considered positive. HER2 status was evaluated using the 2013 ASCO/CAP guidelines after staining by FISH (Fluorescence In Situ Hybridization). Molecular subtypes were identified using the ER and PR status evaluated by IHC and the global HER2 status (IHC + FISH status). A) pHER2Y877 prevalence in the cohort. B) pHER2Y877 distribution according to HER2 status, defined by FISH. (TIF) [file pone.0234991.s002.tif]

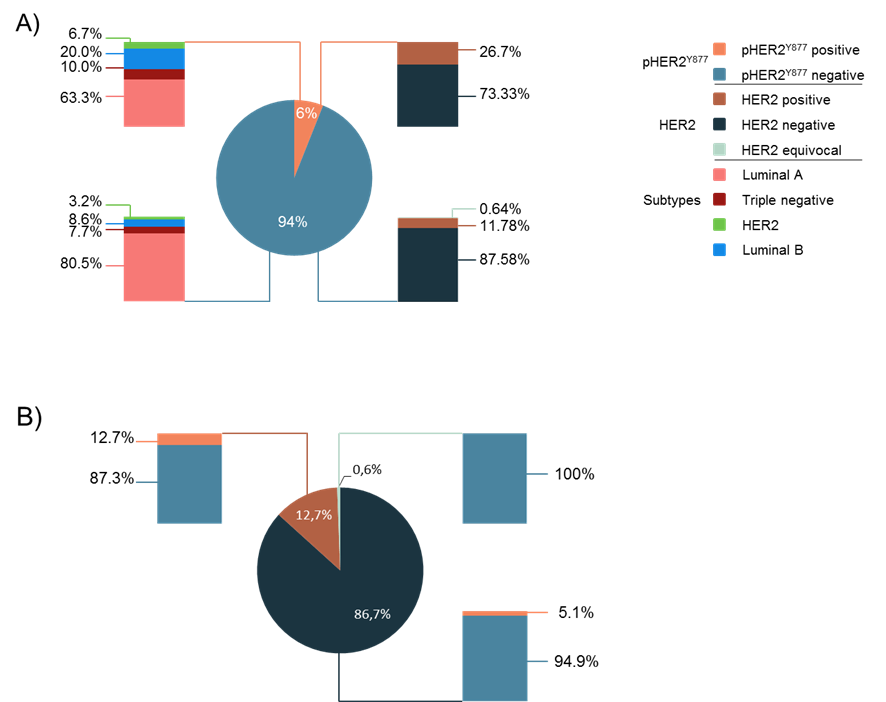

Supplement: S3 Fig — pHER2Y877 status was evaluated using the 2013 ASCO/CAP scoring guidelines after staining by IHC with anti-pHER2Y877 antibody. A score of 2+ pHER2Y877 staining was considered positive. HER2 status was evaluated using the 2013 ASCO/CAP guidelines after staining by FISH and IHC. Molecular subtypes were identified using the ER and PR status evaluated by IHC and the global HER2 status (IHC + FISH status). A) pHER2Y877 prevalence in the cohort. B) pHER2Y877 distribution according to HER2 status, defined by IHC+FISH. (TIF) [file pone.0234991.s003.tif]

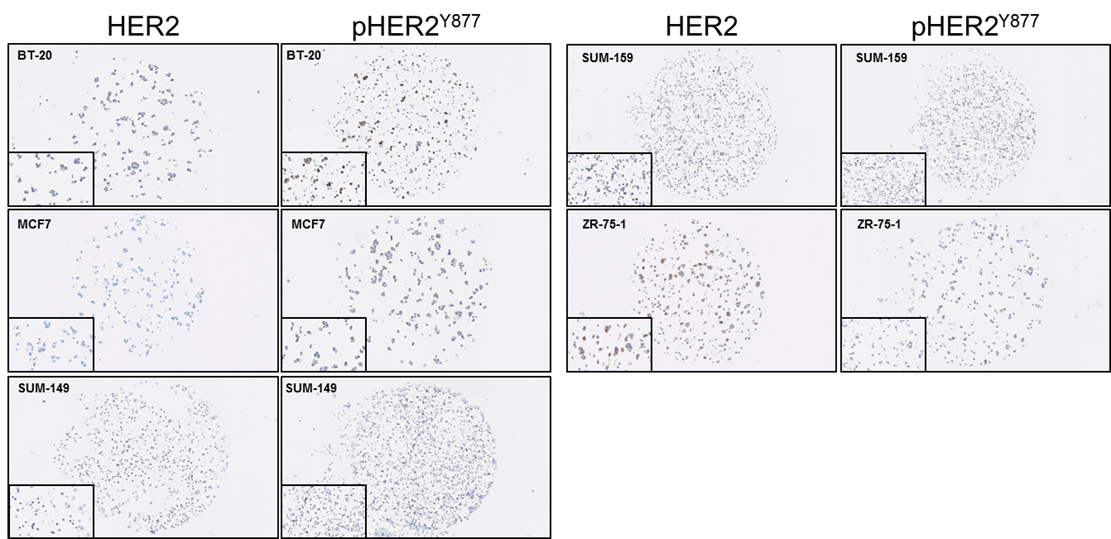

Supplement: S4 Fig — HER2 status was obtained using the HerceptestTM kit–Dako (left column), while pHER2Y877 was performed by IHC with a specific anti-pHER2Y877 antibody (right column). BT-20: HER2-negative; pHER2Y877-positive. MCF7: HER2-negative; pHER2Y877-negative. SUM-149: HER2-negative; pHER2Y877-negative. SUM-159: HER2-negative; pHER2Y877-negative. ZR-75-1: HER2-equivocal; pHER2Y877-negative. (TIF) [file pone.0234991.s004.tif]

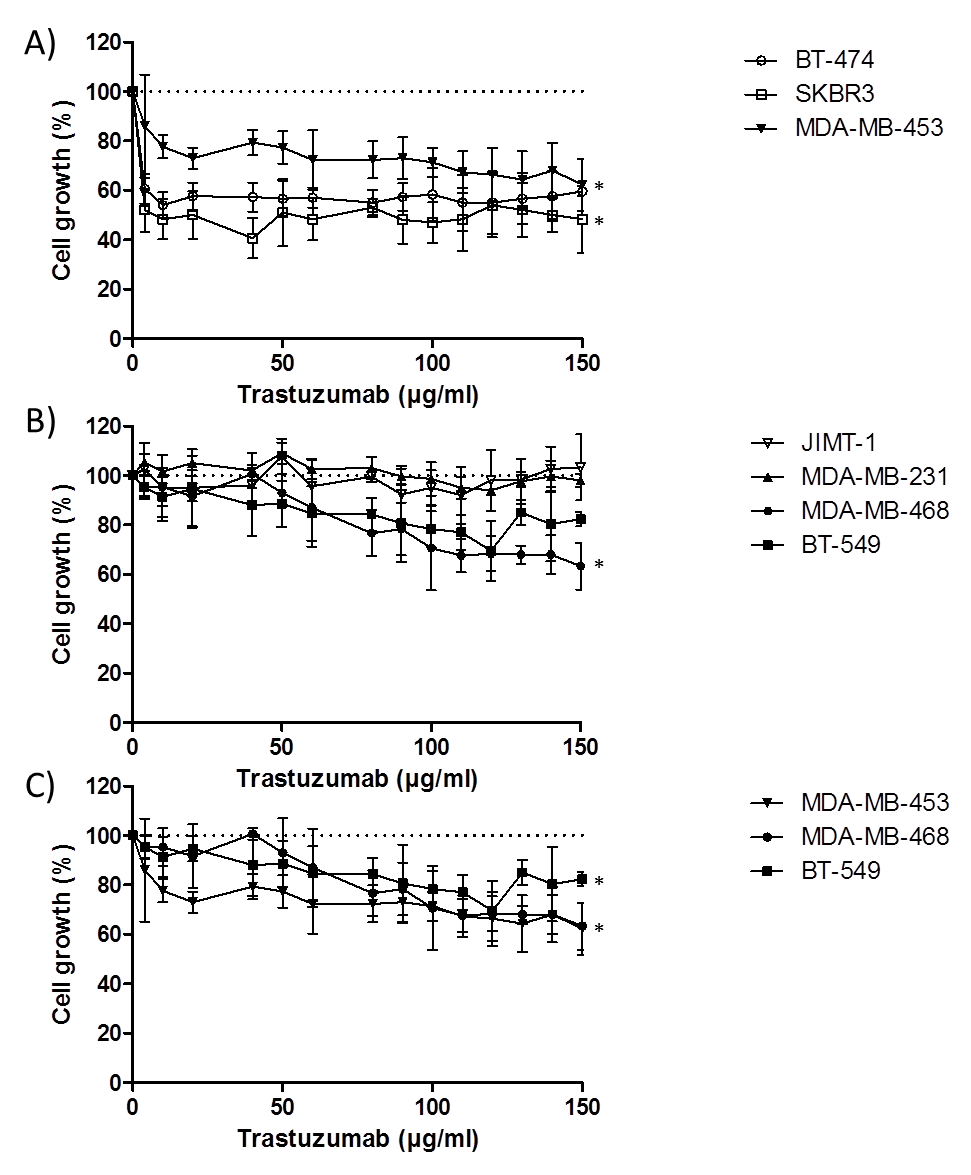

Supplement: S5 Fig — Each cell line was treated with increasing doses of trastuzumab (0, 4, 10, 20, 40, 60, 80, 100, 110, 120, 130, 140 and 150μg/ml). The cell growth was calculated as the percentage of treated cells compared to untreated cells. All experiments were done in triplicates and means ± SD were calculated and plotted for each drug concentration. ANOVA has been done using SAS software. A) Comparison of BT-474 and SKBR3 to MDA-MB-453. B) Comparison of MDA-MB-468 and BT-549 to JIMT-1 and MDA-MB-231. C) Comparison of MDA-MB-468 and BT549 to MDA-MB-453. * p < 0.0001 between cell lines. (TIF) [file pone.0234991.s005.tif]
